# Dutch Dental Hygienists and Their Daily Scope of Practice—A Survey Study

**DOI:** 10.1111/idh.70002

**Published:** 2025-10-02

**Authors:** Meryam Bozia, Erwin Berkhout, Maniesha Shivani Bhagwandat, Fridus van der Weijden, Dagmar Else Slot

**Affiliations:** ^1^ Department of Oral Radiology Academic Centre for Dentistry Amsterdam (ACTA), University of Amsterdam and Vrije Universiteit Amsterdam Amsterdam the Netherlands; ^2^ Department of Periodontology Academic Centre for Dentistry Amsterdam (ACTA), University of Amsterdam and Vrije Universiteit Amsterdam Amsterdam the Netherlands

**Keywords:** dental hygienists, scope of practice, task shifting

## Abstract

**Aim:**

The Dutch government has expanded the status and scope of practice of bachelor's degree dental hygienists (DHs) compared to those with a diploma. The aim of this study is to investigate differences in the daily scope of practice of diploma and bachelor's degree DHs.

**Methods:**

A web‐based survey was emailed to all 2972 members of the Dutch Association of Dental Hygienists and distributed via social media platforms. The survey had sections on participants' demographics, educational qualifications, work environment, and scope of practice. Absolute and relative frequencies for each question were reported and statistically compared between groups.

**Results:**

In total, 473 DHs completed the survey, 288 in the ‘Diploma’ and 185 in the ‘Bachelor’ group. Altogether, Dutch DHs work on average 29 h per week; those with a bachelor's degree work significantly (*p* < 0.001) more hours. Bachelor DHs work significantly (*p* < 0.001) more often as paid employees in a general dental practice, a practice dedicated to periodontology, in the educational and research setting. Diploma DHs work significantly (*p* < 0.001) more often in their own private DH practice. Bachelor DHs are significantly more likely to perform the following activities: administer local anaesthesia, take intra‐oral radiographs, and treat primary caries compared to Diploma DHs (*p* < 0.001).

**Conclusions:**

The overall majority in both groups does not treat caries (81.3%) but does administer local anaesthesia (85.1%). Bachelor DHs are more frequently engaged in three tasks in the extended scope of practice and are more likely to work in a team setting.

## 1 Introduction

The education of dental hygienists (DHs) in The Netherlands has evolved significantly since its inception with a 2‐year curriculum established in 1968 [1]. Developments such as a higher demand for oral healthcare and ongoing task delegation have resulted in an increased workload and led to the extension of the curriculum from two to three years [2], see Online Appendix S1. Regardless of whether a DH completed the 2‐year or 3‐year program, the validity of the Diploma was equal, and it held the same educational value and status. In the clinical practice, there was no discrimination based on educational background or skills among DHs with different training. In 2002, following the Bologna Process and a national shift towards higher education [3], the National curriculum was extended to a four‐year program leading to a Bachelor of Health degree at the University of Applied Sciences. As a result, the first DHs with a bachelor's degree graduated in 2006 [4].

Concurrent with said educational developments, the Dutch government emphasised task shifting and redistribution within the oral healthcare field [5, 6]. This approach was intended to support the ongoing redistribution of tasks between dentists and DHs. This was theoretically supported by educational changes and political discourse to create greater uniformity in the scope of DH practice [2]. The goal was for DHs to assume more responsibilities traditionally held by dentists. However, in later years the Dutch government found that task redistribution between dentists and DHs was still not sufficiently implemented [7]. To address this, the government initiated actively promoting task redistribution through an experiment. For some years, registered DHs will be granted independent authorization to perform certain tasks and activities previously reserved for dentists. These procedures included administering local anaesthesia, taking intra‐oral radiographs, and treating primary caries [8]. DHs have performed these activities for decades already, mostly under supervision or by delegation of a dentist. Understanding how and by whom these activities are performed in clinical practices will help advance and clarify the task redistribution process before the experiment is in full effect.

The most recent comparative study on the scope of practice and educational background of Dutch DHs dates from 2010 [2]. Then, only a small number of DHs with a bachelor's degree and with a few years of experience were practicing, and no major plans for task redistribution were to be expected. Task redistribution in oral health care is a relatively new phenomenon. The Netherlands is a pioneer in the development of independence in the dental hygiene profession [9, 10]. Understanding the differences in practice patterns between diploma and bachelor's degree‐holding DHs can help in workforce planning by addressing shortages in oral healthcare. The aim of this study was to investigate the differences between DHs who have completed a 2‐ or 3‐year diploma curriculum compared to those who have completed the 4‐year bachelor curriculum. The hypothesis is that DHs with a bachelor's degree perform a greater variety and number of activities compared to those with a diploma.

## 2 Materials and Methods

This cross‐sectional observational study and web‐based survey was conducted according to the Consensus‐Based Checklist for Reporting of Survey Studies (CROSS) [11] and the Checklist for Reporting Results of Internet E‐Surveys (CHERRIES) [12].

### 2.1 Ethical Aspects

The Institutional Review Board of the Academic Centre for Dentistry in Amsterdam (ACTA) approved this study under number 202086. The study was conducted according to the code of conduct on health research [13] in accordance with the EU General Data Protection Regulation (GDPR) [14]. The survey was created using SurveyMonkey, a web‐based survey software recognised for its adherence to GDPR regulations [15]. SurveyMonkey utilises Transport Layer Security (TLS) encryption to protect all transmitted information, ensuring that unauthorised access is prevented. Before starting the survey, potential respondents were directed to an online platform hosting a detailed information letter. This letter explained the survey's commitment to maintaining anonymity and provided information about the study's title, background, and objectives. The introduction emphasised the importance of providing honest responses and clarified the purpose of data collection. Participants were then asked to give their consent. If they chose not to consent, the questionnaire was immediately terminated. Since an anonymous survey link was used, personal identifiers such as names and email addresses were not collected. Additionally, SurveyMonkey includes a feature that prevents multiple submissions, which helps to reduce the risk of duplicate entries by securing the survey link [16]. No incentives for participation were offered.

### 2.2 Population

The Dutch Dental Hygienists' Association (NVM‐M) (Nederlandse Vereniging van Mondhygiënisten) is the professional Association for DHs in The Netherlands. The NVM overall policy is to advance the role of DHs in general oral healthcare. To become a full member of the NVM, one must be certified as a DH in the Netherlands, which can be achieved with either a DH Diploma or a DH Bachelor's degree. Therefore, all members of the NVM were eligible and were asked to participate in the survey. Participating was voluntary, and before starting, DHs were informed of the survey's purpose. DH students were not included in the study population. From October to December 2019, registered DHs in The Netherlands were invited to participate in an anonymous online survey. The survey focused on their training, work setting, and common practices related to the administering of local anaesthesia, taking intra‐oral radiographs, and treating primary caries.

The required sample size for this study was estimated using the Sample Size Calculator for Survey Researchers from the website of OvationMR. Based on 4000 dental hygienists in the national dental hygienist licence register in The Netherlands, and assuming a confidence level of 95% with a 5% margin of error, the recommended minimal sample size was 351.

The link to the open, voluntary survey was emailed by the NVM to its 2972 members in October 2019 using a convenience and snowball sample technique. A reminder email was sent to all members after the first email invitation. Moreover, the survey was shared on social media platforms (e.g., Facebook and LinkedIn), and chain referral was used by encouraging DHs to spread the survey link within their networks and non‐members of the NVM.

### 2.3 Questionnaire

The NVM developed the questionnaire using an informal Delphi method based on input from an expert panel of dental hygienist board members and their policy makers with an emphasis on face validity [17]. See Online Appendix S2 for the questions used in Dutch and their English translations. The questionnaire was piloted by a selected group of three experts associated with the Academic Centre for Dentistry in Amsterdam (ACTA) who provided valuable feedback, and amendments were made accordingly. Furthermore, the reliability was assessed by pilot testing in a group of DH members of the NVM. Feedback was used to adapt the questionnaire fully to the Dutch oral health care system, and more working fields were added, such as general practice, own dental hygienist practice, hospital, disabled care, elderly care, public health services (GGD), orthodontics, periodontology, implantology, paediatric, education on different levels, industry, and insurance company.

The questionnaire consisted of closed‐ended and a mix of structured and unstructured questions. It was divided into the following sections: participants' demographics (gender, age, expected age of retirement), educational qualifications (diploma/bachelor, year of graduation), work environment (hours, setting), and scope of practice (focused on administering local anaesthesia, taking intra‐oral radiographs, and treating primary caries). These were the dependent variables. In SurveyMonkey, each page displayed a single questionnaire item. Respondents had the option to review and modify their answers in the system.

### 2.4 Data Organisation

The participants were categorised into two groups: DHs with a Diploma or DHs with a bachelor's degree, and these are the independent variables. The Diploma group contained DHs with a 2‐ and 3‐year curriculum. This group also comprised those who participated in advanced courses for paediatric DH (KTV) (Kindertandverzorgende) or DH with additional skills for adults (MV) (Mondverzorging voor volwassenen) prior to 2002, which focused on preventing and treating caries. The Bachelor group contained those who had completed the 4‐year Bachelor curriculum. DHs who initially pursued a 2‐ or 3‐year curriculum and graduated with a Diploma had the opportunity, during a specific period, to enrol in a two‐year part‐time degree completion program. These DHs who completed this program and obtained a bachelor's degree are included in the Bachelor group. The study also considers other variables, including years since graduation, age, average number of working hours per week, and work settings.

### 2.5 Data Analysis

Only fully completed questionnaires were included in the analysis. All data were automatically exported from the online questionnaire into an Excel data matrix and transferred to SPSS (Statistical Software for the Social Studies, version 26; IBM Corporation, Chicago). The activities performed in the extended scope of practice were nominally measured. Variables like year of birth, year of graduation, weekly working hours, and anticipated retirement age were measured on a ratio level. Participants' demographics and educational qualifications were reported using absolute and relative frequencies for categorical variables and mean and standard deviations for continuous variables. Absolute and relative frequencies of answers for each question were reported and compared between DHs with a Diploma or a bachelor's degree using chi‐square tests for variables with more than two categories. The Mann Whitney *U* test was used for the variables years since graduation and average number of working hours per week. Statistical significance was determined with a threshold value of *p* < 0.05.

## 3 Results

### 3.1 Participants

**FIGURE 1:**
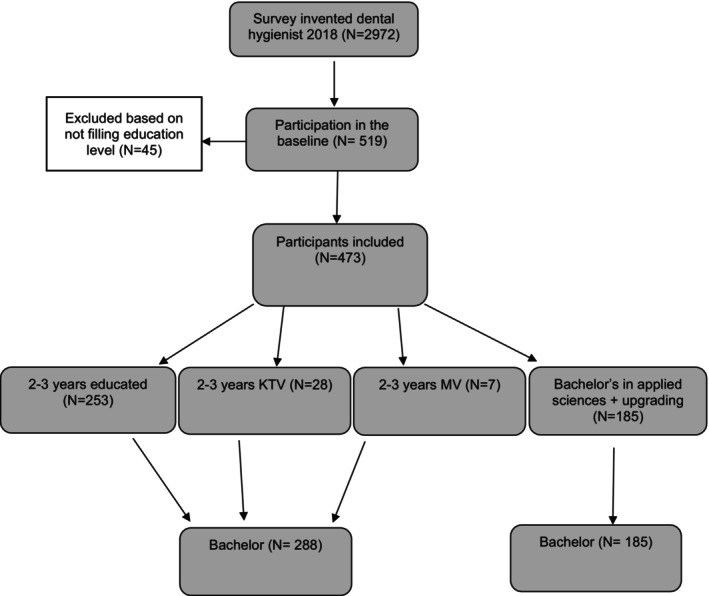
Flowchart of the survey invitation, responses and analysis.

For this study, 2972 DHs and NVM members were invited to complete the questionnaire (see Figure 1). An unknown number of DHs, not being members of the NVM, were reached via social media and chain referral. A total of 519 DHs responded, but 45 did not fully complete the questionnaire. They were excluded due to not answering the question about the level of education. As first, 519 DHs intended to participate, resulting in a response rate of 18%. Finally, 473 DHs were included in the analysis, of which 41 were non‐members of the NVM (8.7%). Of these, 288 were categorised in the Diploma group (2‐ to 3‐year education), and 185 in the Bachelor group (4‐year education). From the Diploma group, 28 respondents completed the postgraduate course for paediatric DHs (KTV), and 7 respondents followed the postgraduate course for DHs with additional skills for adults (MV). The Bachelor group contains 20 respondents who originally graduated with a diploma and followed a degree completion program. See Figure 1 for an overview. As eventually, 473 respondents were included in this study and therefore the calculated ‘a priori’ sample size of 351 was met.

### 3.2 Demographics

Participants demographics are presented in Table 1. In both groups, there were more females (*N* = 456) than males (*N* = 17), and significantly more males (8.1%) in the Bachelor group. The mean age difference between groups was 15 years; the age of the Diploma group (49.2 years) was higher (*p* < 0.001) than the Bachelor group (34.7 years). The mean age at graduation in the Bachelor group (25.1 years) was higher than that of the Diploma group (23.1 years).

**TABLE 1:** Participants demographics.

	Total	Diploma group mean (SD)	Bachelor group mean (SD)	*p*
*N* (%)	473 (100)	288 (60.9)	185 (39.1)	
Gender				
♀female (%)	456 (96.4)	286 (99.3%)	170 (91.9%)	**< 0.001** *
♂male (%)	17 (3.6)	2 (0.7%)	15 (8.1%)	
Age in years	43.5 (11.2)	49.2 (8.3)	34.7 (9.3)	**< 0.001** **
Age range	19–72	30–69	19–72	—
Years graduated	19.8 (11.7)	26.2 ± 8.5	9.7 ± 8.3	**< 0.001** **
Age at graduation	23.7 (4.1)	23.1 ± 3.4	25.1 ± 4.8	**< 0.001** **
Working hours	28.8 (9.0)	27.7 ± 8.3	30.4 ± 9.8	**< 0.001** **
Expected retirement age	62.82 (6.8)	63.7 ± 4.9	61.5 ± 8.9	0.261**

Abbreviation: SD, standard deviation.

* Chi‐square test.

** Mann–Whitney test.

No differences were found for expected retirement age between the groups.

### 3.3 Working Fields and Hours

Dutch DHs worked an average of 29 h per week; those with a Bachelor work significantly more hours (30.4 h) than those with a Diploma (27.7 h) (Table 1). They worked in various fields, with individual DHs potentially working in more than one field. Combined, 473 DHs work in total in 519 fields (see Table 2). None of them worked for the Public Health Service or an insurance company. In six working fields, there was a significant difference (*p* < 0.05) between the Diploma and Bachelor groups. Bachelor DHs work significantly more often as paid employees in a general dental practice, a practice dedicated to periodontology, an independent contractor, or in the educational setting of a University for Applied Sciences or Research University. Diploma DHs worked significantly (*p* < 0.001) more often in their own private DH practice.

**TABLE 2:** Percentage working dental hygienists per field, compared between the diploma and bachelor group.

Working field and setting	Total (*N* = 473) (%)	Diploma group (*N* = 288) (%)	Bachelor group (*N* = 185) (%)	*p* *
Independent practice DH owner	36.8	47.9	36.8	**< 0.001**
General practice. Paid employee	32.3	28.1	38.9	**0.016**
Independent practice. DH self employed	12.3	10.4	15.1	0.151
Self‐employed Other	11.8	8.3	17.3	**0.005**
Elderly care	6.1	4.9	8.1	0.171
Other	5.9	4.9	7.6	0.236
Education Applied Sciences	5.7	3.1	9.7	**0.004**
Employees of independent practice DH	5.1	3.8	5.1	0.136
General practice partner	4.4	5.6	4.4	0.173
Disabled care	3.6	3.5	3.8	1.000
Hospital	3.4	3.5	3.2	1.000
Youth dental care	2.1	1.7	2.1	0.523
Practice periodontology	2.1	1.0	3.8	**0.053**
Education MBO	1.5	1.7	1.1	0.710
Orthodontic practice	1.1	0.7	1.6	0.384
Academic Research	0.8	0.0	2.2	**0.023**
Commerce	0.4	0.3	0.5	1.000
Practice for implantology	0.4	0.0	1.1	0.152
Public health service	0.0	0.0	0.0	—
Health insurer	0.0	0.0	0.0	—

* Fisher Exact test. Bold values, indicate a statistically significant results.

Table 3 is an overview of the hours worked per week by DH groups in different work fields.

**TABLE 3:** Working hours per field, compared between the diploma and bachelor group.

Hours per field	Total (*N* = 473) (mean SD)	Diploma group (*N* = 288)	Bachelor group (*N* = 185)	*p* *
General practice Paid employement	22.2 ± 8.4	21.0 ± 7.3	23.5 ± 9.6	**0.035** *
General practice partner	19.0 ± 10.3	20.3 ± 10.7	14.8 ± 8.4	0.454
Independent practice DH owner	28.0 ± 9.4	28.1 ± 9.0	27.4 ± 10.7	0.712
Employees of independent practice DH	20.6 ± 7.3	18.9 ± 6.1	22.0 ± 8.1	0.208
Independent practice DH self employed	18.1 ± 10.6	15.4 ± 8.5	21.0 ± 11.9	0.065
Independent contracter Other	21.4 ± 11.8	18.1 ± 9.4	23.9 ± 13	0.095
Hospital	16.9 ± 8.3	16.7 ± 10.0	17.2 ± 5.4	0.784
Disabled care	11.2 ± 7.7	9.2 ± 6.8	13.9 ± 8.5	0.254
Elderly care	12.7 ± 8.5	14.1 ± 8.3	11.4 ± 8.8	0.323
Orthodontic practice	14.2 ± 10.0	14.5 ± 2.1	14.0 ± 14	0.564
Practice periodontology	20.0 ± 8.8	23.3 ± 3.1	18.6 ± 10.3	0.168
Practice implantology	24.0 ± 11.3	0	24.0 ± 11.3	—
Youth dental care	23.1 ± 10.9	21.8 ± 14.8	24.4 ± 6.5	0.833
Education HBO	16.5 ± 12.2	10.3 ± 11.0	19.6 ± 11.8	**0.041**
Education MBO	7.1 ± 4.3	7.6 ± 5.2	6.0 ± 1.4	0.693
University Academic Research	13.0 ± 8.9	0	13.0 ± 8.9	—
Commerce	1.0 ± 0	1.0 ± 0	1.0 ± 0	1.0
Other	11.5 ± 7.5	10.7 ± 4.9	12.2 ± 9.5	0.901

*Note:* Bold values, indicate a statistically significant results.

* Mann–Whitney test.

DHs with a bachelor's degree work significantly (*p* < 0.05) more hours than the Diploma DHs in the fields, being employed in a general private practice and in the educational setting of a University for Applied Sciences. No significant difference was found for other work fields between the groups.

### 3.4 Extended Scope of Practice

Regarding the three specific activities within the extended scope of practice, administering local anaesthesia, taking intra‐oral radiographs, and treating primary caries, the Bachelor group differed significantly from the Diploma group. There are more Bachelor DHs that perform these three activities compared to the Diploma DHs. However, specifically for treating caries, the overall majority in both groups do not treat caries (81.3%), see Table 4.

**TABLE 4:** The extended scope of practice compared between the groups.

Ex. task		Total *N* (%)	Diploma group *N* (%)	Bachelor group *N* (%)	*p* *
Local anaesthesia	Yes	400 (85.1)	225 (78.9)	175 (94.6)	**< 0.001**
	No	70 (14.9)	60 (21.1)	10 (5.4)	
X‐rays	Yes	271 (57.7)	142 (49.8)	129 (69.7)	**< 0.001**
	No	199 (42.3)	143 (50.2)	56 (30.3)	
Caries preperation	Yes	88 (18.7)	20 (7.0)	68 (36.8)	**< 0.001**
	No	382 (81.3)	265 (93.0)	117 (63.2)	

* Chi‐square test.

The analysis performed on the three activities in relation to years of graduation and working hours per week did show some differences. In the Diploma group, DHs who do not administer local anaesthesia graduated longer ago than those who do administer it (*p* < 0.001). In the Bachelor group, DHs who do not take intra‐oral radiographs graduated a longer time ago than those who do take them (*p* < 0.001). In the Diploma group, DHs who do not take intra‐oral radiographs do work more hours compared to DHs who do take intra‐oral radiographs (*p* < 0.001). No difference was found for the activity caries treatment when related to the year of graduation or working hours for both groups; see Table 5.

**TABLE 5:** Extended scope of practice related to working hours per week and years since graduation.

Group	Ex. task	*N*	Hours a week (mean)	*p* *	Graduated years (mean)	*p* *
Diploma group	Anaesthesia	Yes 225	28.0	0.521	25.0	**< 0.001**
		No 60	26.8		31.0	
Bachelor group	Anaesthesia	Yes 175	30.8	0.186	9.4	0.394
		No 10	24.2		15.2	
Diploma group	X‐ rays	Yes 142	26.1	**< 0.001**	26.3	0.960
		No 143	29.4		26.2	
Bachelor group	X‐rays	Yes 129	31.1	0.318	8.8	**< 0.001**
		No 56	28.8		11.8	
Diploma group	Caries preperation	Yes 20	26.5	0.495	26.3	0.845
		No 265	27.9		26.2	
Bachelor group	Caries preperation	Yes 68	30.5	0.730	9.4	0.461
		No 117	30.4		9.9	

* Mann–Whitney *U* test.

## 4 Discussion

### 4.1 Work Activities

Work activities between the DHs groups were compared. The hypothesis was that Bachelor DHs perform more activities than Diploma DHs in the extended scope of practice. This hypothesis was confirmed by the research findings; more Bachelor DHs perform the activities of administering local anaesthesia, taking intra‐oral radiographs, and treating primary caries. In 2002, the education program for DHs was expanded to include competencies in diagnosing and treating caries [18]. A study from 2010, conducted just after the newly educated DHs entered the workforce, showed that DHs with a bachelor's degree had a significantly different scope of practice in terms of caries diagnosis and treatment [2]. Despite these DHs being young and having less work experience, their education resulted in them having an extended scope of practice. This aligns with the present findings for the Bachelor DHs regarding treating caries. Although previous research found no significant differences in the frequencies of performing activities associated with anaesthesia and intra‐oral radiographs between Diploma and Bachelor DHs, the present study does report a difference for these activities [2]. These findings could be explained by increased work experience since 2009 and further task redistribution acceptance in the field. In the Bachelor group, the mean years since graduation is 3 times (9.7 years) as much as in previous research, where the maximum of years since graduation was 3 years.

### 4.2 Demographics of the Dental Hygiene Profession

The research findings show that DHs with a Diploma exhibited a notably higher average age when compared to those in the Bachelor group. The latter group graduated from the year 2006 onwards. The age at graduation in the Bachelor group was higher. This can logically be explained by the duration of the Diploma degree that was 2–3 years and the bachelor's degree that is 4 years. Furthermore, the results indicate a statistically higher prevalence of DHs with a bachelor's degree among men. Prior research [19] anticipated an increase in male representation within healthcare professions such as nursing and dental hygiene, yet the DH field remains predominantly female [20]. The change towards a bachelor's degree with a focus on certain activities and responsibilities has resulted in a different professional status. The extended scope of practice including curative activities potentially makes the profession more attractive to males, compared to a profession focused primarily on oral disease prevention. The results show that Bachelor DHs work more hours than Diploma DHs. This could be due to them being younger and not being involved in the overall tradition of working part‐time after having children [21]. Having more males in the Bachelor group presumably contributes to more working hours as well [22, 23].

### 4.3 Work Setting

Earlier research concluded that almost half of Diploma DHs run their own private practice [24]. It was explained that they create an intrinsically rewarding job by accepting a restricted scope of practice but compensate for this with self‐employment in a mono‐disciplinary setting. This could explain present research findings that many DHs in the Bachelor group are employed in larger team settings within a general dental practice, whereas those in the Diploma group more often operate their own private practices. This implicates that Bachelor DHs like to work according to the extended scope of practice in a clinical team but not as practice owners. Other reasons for not owning a private practice could be lack of experience, insurance reimbursement challenges, and start‐up costs [25]. These are greater thresholds for young Bachelor DHs than older Diploma DHs. This could also explain why Bachelor DHs work significantly more often in educational settings and no Diploma DHs worked in a research setting. This may further be explained by the requirements that a master's degree is often needed to serve as a teacher at universities where Bachelor DHs are educated. Additionally, a master's degree is mandatory for entering a doctorate program [26]. This implies that only Bachelor DHs or those with additional education are eligible for these positions at university and for academic research settings.

Literature has stated that motivating factors such as responsibility, job variation, and recognition contribute positively to engagement among oral health professionals [27, 28]. This implicates that the position of DHs with diplomas may be influenced or even challenged if those with a bachelor's degree assume different responsibilities in oral health care and the clinical practice due to altered legal status through task redistribution. The NVM advocates for all DHs, regardless of their current training, to have their additional training recognised under a different section of Dutch law, enabling them to take on expanded tasks and increased responsibilities. Despite a nationwide shift in the education system from diplomas to bachelor's degrees, the NVM advocates that the legal status change should be possible for all Dutch DHs regardless of their education. However, the Royal Dutch Dental Association (KNMT) supports granting a distinct legal status, along with specific tasks and responsibilities, exclusively to those with a bachelor's degree. These dichotomous perspectives hinder further task redistribution in oral healthcare and contribute to unequal access to oral health care for patients.

### 4.4 Implications for Education and Clinical Practice

Numerous factors impact the effectiveness of task redistribution, including alterations in educational and legislative policies, as well as interconnected organisational and individual factors [29, 30, 31]. The findings indicate that DHs with bachelor's degrees are more likely to engage in extended scope of practice and work in collaborative settings. This underscores the importance of promoting higher education within the profession. Ongoing professional development is crucial for dental hygienists, especially those with diplomas who may need additional training to perform extended scope activities. Therefore, continuing education programs should be available to help diploma‐holding DHs upgrade their skills and knowledge, ensuring they remain competitive and capable of meeting the demands of modern dental practice. The findings that bachelor's degree‐holding dental hygienists are more likely to work in collaborative settings highlight the importance of interprofessional education. Interprofessional education prepares the future professional for the efficient use of the right care, where everyone's roles and responsibilities are utilized to improve health outcome [32]. Dutch researchers have noticed that in daily practice “novice” dentists and dental hygienists experienced interprofessional collaboration to be in its early development [33] and further encouragement is needed. Dental hygiene programs should include more opportunities for students to learn and practice alongside other healthcare professionals such as prevention or prophylaxis assistants, fostering teamwork and improving integrated care delivery.

Practical implications of this study are relevant for policymakers, educators, and practitioners aiming to optimise the utilisation of DHs in oral healthcare. Clinical practices can use this information to develop targeted recruitment and training strategies, ensuring a well‐distributed and adequately skilled dental hygiene workforce. If a clinical practice requires a professional with a primarily preventive focus who prefers to work independently, emphasis should be placed on employing a diploma‐qualified DH. Conversely, if there is a need for a care provider with a more curative orientation who is open to interdisciplinary collaboration, preference should be given to a DH with a bachelor's degree.

### 4.5 Strength and Limitations

During the 2019 survey period, the National Licence Register reported an estimated 4000 graduated DHs in the Netherlands, with roughly half of them holding a Diploma and the other half holding a bachelor's degree [34]. The questionnaire was distributed to all members of the NVM and achieved a robust sample size, ensuring a broad and representative sample of the population. Nonetheless, the response rate among DHs with a Diploma was higher than that for DHs with a bachelor's degree. This may be attributed to the relatively older DHs with Diplomas placing greater importance on the research topic due to their entrepreneurial roles. Older DHs, predominantly diploma holders, aim to safeguard their professional status, rooted in the early development of the DH profession. They have played a pivotal role in formalising the DH role, beginning in 1967, actively participating and maintaining a strong professional identity and relationship with the DH association [18].

Because of the anonymity of the study set up, asking for clarifications about answers was not possible. Adhering to GDPR regulations, however, ensured the integrity of the research process and the protection of participants' rights. This way, information was gathered about what the situation was before the experiment of task redistribution started. The study fills a gap in the existing literature by providing updated research on the scope of practice of Dutch dental hygienists, which had not been comprehensively studied since 2010. This contribution is significant for understanding the evolution of the profession and the impact of educational reforms and expected task redistribution.

A limitation is that the study relied on self‐reported data, which can be subject to recall bias and social desirability bias. Participants might have overestimated or underestimated their scope of practice and work hours, affecting the accuracy of the results. As a cross‐sectional study, it captures data at a single point in time, limiting the ability to infer causality or changes over time. Longitudinal studies would be needed to observe trends and the long‐term impact of educational and political changes on the scope of practice. Including qualitative data through interviews or open‐ended survey questions could provide deeper insights into the challenges and benefits of task redistribution, especially with further expected task redistribution in the future.

### 4.6 Implications for Future Research

The findings of this study indicate the potential for increased utilisation of extended activities and the possibility of enhanced DH education to a bachelor's degree has partially aided in DHs performing more activities in the extended scope of practice. Although we have yet to see DHs use their full capabilities to treat caries. The expansion of the DH education program was, among other reasons, implemented to enable DHs to treat caries more frequently through task redistribution. The DH and the dental educational institutions should discuss how to improve future DHs' participation in caries treatment activities based on the results. Since task redistribution is an ongoing process, and the data were collected before the current governmental experiment in the Netherlands, further evaluation on this topic and comparative variables in a few years is strongly recommended. The same recommendation applies to research in other EU countries. Although there is a common framework for DHs in Europe [35], the literature shows that the practice of DHs is structured and regulated differently across countries, which accounts for differences.

## 5 Conclusion

There are notable differences between Diploma and Bachelor DHs. In summary, Bachelor DHs engage more frequently in the extended scope of practice and work more often in collaborative team settings with other professionals. Regulatory bodies may consider revising scope of practice laws to reflect the competencies of bachelor's degree‐holding dental hygienists, facilitating greater task redistribution from dentists to dental hygienists.

## 6 Clinical Relevance

### 6.1 Scientific Rationale for the Study

There is a need for a better understanding of and comparison of the extended scope of practice and educational background of dental hygienists. Dutch dental hygienists are bound to receive more legal independence in their profession.

### 6.2 Principal Findings

There are more bachelor educated dental hygienists that perform activities in the extended scope of practice. These tasks are meanly administrating local anaesthesia, taking intra‐oral radiographs and less so treating primary caries compared to diploma educated dental hygienist.

### 6.3 Practical Implications

The transition of dental hygiene education to a bachelor's degree has partially aided in dental hygienists performing more activities in the extended scope of practice.

## Author Contributions

All authors gave their final approval and agreed to be held accountable for all aspects of the work, ensuring integrity and accuracy. M.B.: contributed to conception, design, analysis, and interpretation, and drafted the manuscript. E.B.: contributed to interpretation and critically revised the manuscript. M.S.B.: contributed to conception, design, analysis, and interpretation and drafted the preliminary version of the manuscript. F.W.: contributed to interpretation and critically revised the manuscript. D.E.S.: contributed to conception and design, analysis, interpretation, and critically revised the manuscript.

## Conflicts of Interest Statement

The authors declare no conflicts of interest. Slot is a dental hygienist, Bhagwandat is a dental hygienist and dentist, and Bozia, Berkhout, and van der Weijden are dentists.

## Supporting information


**Data S1:** idh70002‐sup‐0001‐Supinfo1.pdf.

## Data Availability

The data that support the findings of this study are available from the corresponding author upon reasonable request.
