# Supplementary material for: Dutch Dental Hygienists and Their Daily Scope of Practice—A Survey Study
Source: Int J Dent Hyg. 2025 Oct 2;24(2):216–25. doi: 10.1111/idh.70002 (PMC13050384; doi:10.1111/idh.70002)
Supplement: Supplementary file 1 — Data S1: idh70002‐sup‐0001‐Supinfo1.pdf. [file IDH-24-216-s001.pdf]

- 1920 — **Introduction** *oral care professional* in the Netherlands
- 1968 — **First school** for *dental hygiene in faculty of dentistry in Utrecht*
- 1970s — First experiments on task distribution from dentist to dental hygienst
- 1974 — Legal recognition Law of Paramedical professions (*Wet op de Paramedische beroepen)*; introduction of Dental Hygienist Resolution
- 1985 — Report from the Committee for educational advise for Dentist (adviescommissie Opleiding Tandarts - AOT) *Pleaded for adequate teamwork education for dentist which would reduce the number of required dentist, at organizational level optimal cooperational was hard to realize because dentist were not educated to work together with dental hygienist*
- 1968-1992 — **Two-year** curriculum Dutch dental hygiene
- 1992-2002 — **Three-year** curriculum Dutch dental hygiene
- 1992 — Ministry iniated the development of the profile of the *dental hygiene profession*
- 1994 — *Dental hygienist working under instruction and control of dentist* **changed** to *dental hygienst working with dentist*
- 1997 — The *Market Competition and the Pricing Proces in Healthcare* **report** reopend discussions about task distribution in dental healthcare. This report reopend discussions about task distribution in dental healthcare
- 1997 — **BIG-law** (Wet op de Broepen in de Individuele Gezondheidszorg) was introduced
- 2000 — The Lapré Committee was formed to investigate the nature, gravity and magnitude of de capacity shortage in oral healthcare and make recommendations to address the shortage (The Committee for Capacity in Oral Healthcare)
- 2002-present — **Four-year** curriculum Dutch dental hygiene
- 2006 — Corresponding change in legisation regarding the *functional independacy* and *free accessibility* of dental hygienist by ministery VWS
- 2006 — *The innovation in Oral Healthcare Committee underlinded various possibilitis for dental hygienst to take over routine tasks from dentist.*
- 2009 — **Capacityplan 2009 (Capaciteitsplan 2009: Mondzorg) report**
- 2010 — **Capacityplan 2010 (Capaciteitsplan 2010: Deelrapport 3 Mondzorg) report**
- 2013 — **Capacityplan 2013(Capaciteitsplan 2010: Deelrapport 3 Mondzorg) report**
- 2019 — **Maastricht UMC+ report** inventarisation vertical task shiftng first line dental care 2015-2018
- 2019 — **Report** client research first line dental care commissioned by the Advisory Committee
- 2020 — Nivel 2020 **report** supply dental care
- april 2020 — Registration Temporary Register by dental hygienist to independenly execute tasks
- Juli 2020 — Start five year experiment to execute tasks independenly

Appendix 1 – Timeline

Appendix 2 - Questionnaire

Enquête om gegevens te verkrijgen voor Capaciteitsorgaan 2018 (eigendom van het NVM)

Inleiding

Naar aanleiding van alle vragen rondom capaciteit in de mondzorg en het rapport van Panteia dat niet heeft opgeleverd wat gehoopt, heeft minister Bruno Bruins besloten om aan het Capaciteitsorgaan te vragen een nieuwe capaciteitsraming uit te voeren. Daarbij is het van belang dat wij zoveel mogelijk actuele gegevens hebben over de beroepsuitoefening van mondhygiënisten. Wij willen u dan ook vragen ons te helpen en de vragen uit deze enquête te beantwoorden. Het Capaciteitsorgaan stelt een advies op aan de hand van deze gegevens en volgens de systematiek die het Capaciteitsorgaan hanteert voor het opstellen van een dergelijk instroomadvies, ten behoeve van het Ministerie van OC&W.

Er zijn ook enkele vragen over kindertandverzorging en mondverzorging voor volwassenen opgenomen. Dit zijn twee post-hbo opleidingen die in de jaren voor 2002 (start 4-jarige opleiding) zijn aangeboden aan mondhygiënisten. Deze post-hbo opleidingen voor mondhygiënisten zijn beide in Nijmegen gegeven. Heeft u deze opleidingen niet gevolgd, dan hoeft u deze vragen niet verder te beantwoorden.

Voorstel vragen:

Bent u lid van de Nederlandse Vereniging van Mondhygiënisten? - Ja

- Nee

Wat is uw geslacht? - Vrouw

- Man

Wat is uw geboortejaar ?
In welk jaar bent u afgestudeerd?

Welke opleiding heeft u gevolgd?

- - 2-jarige
- - 3-jarige
- - 4-jarige – ga naar vraag .....

Werk u momenteel voltijds of in deeltijd als mondhygiënist in Nederland?

- - Ja, ik werk voltijds
- - Ja, ik werk in deeltijd, namelijk .. fulltime equivalenten (FTE)

[Toelichting: vier dagen per week is 0,8 FTE, drieënhalve dag is 0,7 FTE, drie dagen is 0,6 FTE et cetera]

- - Nee, ik ben momenteel werkzaam buiten Nederland
- - Nee, ik ben niet meer werkzaam als mondhygiënist sinds .... [jaar]

En hoeveel uur per week bent u gemiddeld op dit moment per week werkzaam als mondhygiënist in Nederland?

Gemiddeld ... uur per week
Geef per werkveld aan hoeveel uren u daarin werkzaam bent per week (meerdere antwoorden mogelijk)

- - Algemene praktijk in loondienst
- - Algemene praktijk, meewerkend partner
- - Mondhygiënepraktijk vrije vestiging
- - Mondhygiënepraktijk in loondienst
- - Mondhygiënepraktijk werkend als ZZP
- - Andere praktijk setting (geen MH-praktijk) werkend als ZZP
- - Ziekenhuis
- - Gehandicaptenzorg
- - Ouderenzorg
- - GGD
- - Orthodontiepraktijk
- - Parodontologiepraktijk
- - Implantologiepraktijk
- - Jeugdtandverzorging
- - Onderwijs HBO
- - Onderwijs MBO
- - Onderzoek
- - Commercie
- - Zorgverzekeraar
- - Overige

Tot welke leeftijd denkt u te werken als mondhygiënist in Nederland?

- Tot .. jaar

Heeft u in uw huidige werk klinisch contact met patiënten?

- - Ja, ga naar vraag ....
- - Nee, dan stopt hier de enquête

Heeft u de aanvullende opleiding Kindertandverzorgende (KTV) in Nijmegen gevolgd?

- Ja

Zo ja, voert u de volgende voorbehouden handelingen uit? ○ Geven van anesthesie

○ Ja ○ Nee

Zo nee, waarom niet?

○ Maken en interpreteren röntgenfoto’s ○ Ja

○ Nee
Zo nee, waarom niet?

○ Behandeling primaire caviteiten ○ Ja

○ Nee
Zo nee, waarom niet?

- Nee

Heeft u de aanvullende opleiding Mondverzorging voor Volwassenen (MV) in Nijmegen gevolgd?

- Ja
Zo ja, voert u de volgende voorbehouden handelingen uit?

○ Geven van anesthesie ○ Ja

○ Nee
Zo nee, waarom niet?

○ Maken en interpreteren röntgenfoto's ○ Ja

○ Nee
Zo nee, waarom niet?

○ Behandeling primaire caviteiten ○ Ja

○ Nee
Zo nee, waarom niet?

- Nee

Als u in eerste instantie 2- of 3-jarig bent opgeleid, heeft u de upgrading voor bachelor gevolgd en afgerond?

- Ja, ga naar vraag..... - Nee

Als u 2- of 3-jarig bent opgeleid (zonder upgrading of aanvulling KTV of MV), voert u de volgende voorbehouden handelingen uit?

- - Geven van anesthesie
    - Ja
    - Nee
      Zo nee, waarom niet?
- - Maken en interpreteren röntgenfoto's
    - Ja
    - Nee
      Zo nee, waarom niet?
- - Behandeling primaire caviteiten
    - Ja
    - Nee
      Zo nee, waarom niet?

Als u
- Geven van anesthesie

4-jarig bent opgeleid, voert u de onderstaande voorbehouden handelingen uit?

- Ja
- Nee

  Zo nee, waarom niet?

- - Maken en interpreteren röntgenfoto's
  - Ja
  - Nee

    Zo nee, waarom niet?

- - Behandeling primaire caviteiten
  - Ja
  - Nee

    Zo nee, waarom niet?

Als u 4-jarig bent opgeleid, bent u dan van plan mee te doen met het experiment m.b.t. de volledige zelfstandige bevoegdheid voor de drie voorbehouden handelingen (anesthesie, röntgen, behandeling primaire caviteiten)?

- Ja
Zo ja, welke van de voorbehouden handelingen verwacht u uit te gaan voeren • Anesthesie
• Röntgen
• Behandeling primaire caviteiten

- Nee
Zo nee, waarom niet?

Als er een mogelijkheid komt voor de 2- en 3-jarig opgeleiden om in het experiment in te stromen door het afnemen van een toets om eerder verworven competenties aan te tonen, doet u dan mee met het experiment?

- Ja
Zo ja, welke van de voorbehouden handelingen verwacht u uit te gaan voeren • Anesthesie
• Röntgen
• Behandeling primaire caviteiten

- Nee
Zo nee, waarom niet?

Bent u van plan de upgrading voor bachelor te volgen, als u daarmee in kunt stromen in het experiment?

- Ja
- Nee
